# Impact of clonal lineages on susceptibility of *Staphylococcus lugdunensis* to chlorhexidine digluconate and chloride benzalkonium

**DOI:** 10.1186/s12866-023-03088-1

**Published:** 2023-11-13

**Authors:** Laurie Destruel, Marine Lecomte, Maxime Grand, Marie Leoz, Martine Pestel-Caron, Sandrine Dahyot

**Affiliations:** 1 Univ Rouen Normandie, UNICAEN, Inserm, Normandie Univ, DYNAMICURE UMR 1311, F - 76000 Rouen, France; 2 Univ Rouen Normandie, UNICAEN, Inserm, Normandie Univ, DYNAMICURE UMR 1311, CHU Rouen, Department of Bacteriology, F - 76000 Rouen, France

**Keywords:** *Staphylococcus lugdunensis*, Chlorhexidine digluconate, Benzalkonium chloride, Resistance, Antiseptics, MIC/MBC values, *qac*, *nor*A

## Abstract

**Background:**

Little is known about susceptibility of *Staphylococcus lugdunensis* to antiseptics. The objective of this study was to evaluate, at the molecular and phenotypic level, the susceptibility of 49 clinical *S. lugdunensis* strains (belonging to the seven clonal complexes [CCs] defined by multilocus sequence typing) to two antiseptics frequently used in healthcare settings (chlorhexidine digluconate [CHX] and chloride benzalkonium [BAC]).

**Results:**

The minimum inhibitory concentrations (MICs), by broth microdilution method, varied for BAC from 0.25 mg/L to 8 mg/L (MIC_50_ = 1 mg/L, MIC_90_ = 2 mg/L) and for CHX from 0.5 mg/L to 2 mg/L (MIC_50_ = 1 mg/L, MIC_90_ = 2 mg/L). The BAC and CHX minimum bactericidal concentrations (MBCs) varied from 2 mg/L to 8 mg/L (MBC_50_ = 4 mg/L, MBC_90_ = 8 mg/L) and from 2 mg/L to 4 mg/L (MBC_50_ and MBC_90_ = 4 mg/L), respectively. A reduced susceptibility to CHX (MIC = 2 mg/L) was observed for 12.2% of the strains and that to BAC (MIC ≥ 4 mg/L) for 4.1%. The *norA* resistance gene was detected in all the 49 isolates, whereas the *qacA* gene was rarely encountered (two strains; 4.1%). The *qacC*, *qacG*, *qacH*, and *qacJ* genes were not detected. The two strains harboring the *qacA* gene had reduced susceptibility to both antiseptics and belonged to CC3.

**Conclusion:**

The *norA* gene was detected in all the strains, suggesting that it could belong to the core genome of *S. lugdunensis*. *S. lugdunensis* is highly susceptible to both antiseptics tested. Reduced susceptibility to BAC and CHX was a rare phenomenon. Of note, a tendency to higher MICs of BAC was detected for CC3 isolates. These results should be confirmed on a larger collection of strains.

**Supplementary Information:**

The online version contains supplementary material available at 10.1186/s12866-023-03088-1.

## Background

*S. lugdunensis* is a particularly virulent species of coagulase-negative staphylococci (CoNS) [1]. This microorganism is a skin commensal but is also an opportunistic pathogen able to cause potentially severe community-acquired infections as well as healthcare-associated infections [2, 3].

Several molecular typing methods, including multilocus sequence typing (MLST) and *fbl*-typing, revealed a clonal population structure, with no phylogenetic lineage associated with invasive infections [4, 5]. To date, seven clonal complexes (CCs) have been described (http://bigsdb.web.pasteur.fr/staphlugdunensis/). Strains isolated worldwide from hospitalized patients mainly belong to CC1 and CC3 [6–8]. Even if some methicillin resistant clones are circulating in Asia [8–10], this species currently remains highly sensitive to antibiotics [3]. Predominance of some CCs could then be explained by the low genetic diversity of the species [11] or by an increased ability of these CCs to survive in the hospital environment by biofilm formation [1] and/or resistance to antiseptics.

Two antiseptics, chlorhexidine digluconate (CHX) and benzalkonium chloride (BAC), are widely used in health care settings including skin and wound antisepsis [12, 13]. Antiseptic resistance of *Staphylococcus aureus* and CoNS is mediated by efflux pumps: NorA and QacA belonging to the major facilitator superfamily, and QacC, QacG, QacH and QacJ belonging to the multidrug resistant transporter family [14–16]. This resistance is phenotypically characterized by high minimum inhibitory concentrations (MICs) to antiseptics, even if there is no consensual definition [17, 18]. Antiseptic resistance can be associated with the presence of resistance genes, which can belong to the core genome such as the *norA* gene in *S. aureus* [19], or be encoded by a plasmid like *qac* genes, identified in *S. aureus* and various species of CoNS [14].

So far, little is known about *S. lugdunensis* resistance to antiseptics [20, 21]. Therefore, the aim of this study was to investigate the prevalence of resistance to the widely used antiseptics BAC and CHX by using both phenotypic and genotypic methods, in a collection of *S. lugdunensis* clinical isolates representing the seven CCs described to date.

## Results

### Antimicrobial susceptibility test results

The antibiotic susceptibility results of the 49 *S. lugdunensis* strains are shown in Table 1. Eleven strains (22.4%) were resistant to penicillin G. The rate of strains resistant to kanamycin, tobramycin, fusidic acid and tetracyclin was 4.1% (2/49 strains) and that of strains resistant to gentamicin, erythromycin and clindamycin was 2% (1/49 strains). One strain (2%) was resistant to methicillin (methicillin-resistant *S. lugdunensis*, MRSL). This strain was multidrug resistant and belonged to CC3.

**Table 1:** Antibiotic susceptibility testing for the 49 *S. lugdunensis* strains

Antibiotic (49 strains)	Susceptible No. (%)	Resistant No. (%)
penicillin G	38 (77.6)	11 (22.4)
kanamycin	47 (95.9)	2 (4.1)
tobramycin	47 (95.9)	2 (4.1)
gentamicin	48 (98)	1 (2)
erythromycin	48 (98)	1 (2)
clindamycin	48 (98)	1 (2)
quinupristin/ dalfopristin	49 (100)	0 (0)
cefoxitin	48 (98)	1 (2)
norfloxacin	49 (100)	0 (0)
trimethoprim/ sulphamethoxazole	49 (100)	0 (0)
fusidic acid	47 (95.9)	2 (4.1)
rifampicin	49 (100)	0 (0)
levofloxacin	49 (100)	0 (0)
tetracyclin	47 (95.9)	2 (4.1)
minocyclin	49 (100)	0 (0)
linezolid	49 (100)	0 (0)

### Determination of minimum inhibitory concentration and minimum bactericidal concentration

The MICs of CHX ranged from 0.5 to 2 mg/L and those of BAC from 0.25 to 8 mg/L (Table 2). The MIC_50_ and MIC_90_ were 1 mg/L and 2 mg/L respectively, for both CHX and BAC. Reduced susceptibility to CHX and BAC was observed for 12.2% (6/49) and 4.1% (2/49) of strains, respectively.

**Table 2:** Susceptibility testing of the 49 *S. lugdunensis* strains to CHX and BAC

Antiseptic resistance genes in strains (*n* = 49)	CHX MIC (mg/L)				BAC MIC (mg/L)					
	0.5	1	2		0.25	0.5	1	2	4	8
*norA* (*n* = 49)	1	42	**6**		2	3	24	18	**1**	**1**
*qacA* (*n* = 2)	0	0	2*		0	0	0	0	1*	1*
*qacC/qacG/qacH/qacJ* (*n* = 0)	0	0	**0**		0	0	0	0	**0**	**0**

CHX, chlorhexidine digluconate; BAC, benzalkonium chloride; *Clonal complex 3.

Strains with a reduced susceptibility are in bold text.

The minimum bactericidal concentrations (MBCs) ranged from 2 to 4 mg/L and 2 to 8 mg/L for CHX and BAC, respectively. The MBC_50_ and MBC_90_ of CHX were 4 mg/L. The MBC_50_ and MBC_90_ of BAC were 4 mg/L and 8 mg/L, respectively.

MIC and MBC distribution of CHX and BAC according to the CC is shown in Table 3. BAC MICs tended to vary according to the CC of the strains. The BAC MICs of the CC5 and singleton (belonging to any of the CC described) strains (mean of 0.55 mg/L and 0.81 mg/L, respectively) appeared to be lower than those of the other CCs. On the contrary, strains belonging to CC3 had the highest MIC values (mean of 2.18). No noticeable trend was highlighted regarding the MBC distributions of either CHX or BAC.

**Table 3:** MIC and MBC values of CHX and BAC according to the CC

Clonal Complex (*n* = 49 strains)		CHX					BAC			
		MIC range (mg/L)	MIC mean (mg/L) (SEM)	MBC range (mg/L)	MBC mean (mg/L) (SEM)		MIC range (mg/L)	MIC mean (mg/L) (SEM)	MBC range (mg/L)	MBC mean (mg/L) (SEM)
1 (*n* = 11)		1–2	1.27 (0.14)	2–4	0.82 (0.30)		1–2	1.45 (0.16)	2–4	3.82 (0.18)
2 (*n* = 5)		1	1.00 (0)	2–4	3.60 (0.40)		1–2	1.60 (0.24)	2–4	3.60 (0.40)
3 (*n* = 11)		1–2	1.18 (0.12)	2–4	2.91 (0.31)		1–8	2.18 (0.64)	4–8	5.09 (0.56)
4 (*n* = 4)		1	1.00 (0)	2–4	3.00 (0.58)		1–2	1.50 (0.29)	4–8	5.00 (1.00)
5 (*n* = 5)		1–2	1.20 (0.2)	2–4	2.80 (0.49)		0.25-1	0.55 (0.12)	2–4	3.60 (0.40)
6 (*n* = 5)		1	1.00 (0)	2–4	3.20 (0.49)		1–2	1.60 (0.24)	4–8	5.6 (0.98)
7 (*n* = 4)		1	1.00 (0)	2–4	3.50 (0.50)		1–2	1.50 (0.29)	4–8	5 (1.00)
singleton ST (*n* = 4)		0.5-1	0.88 (0.13)	2–4	3.00 (0.58)		0.25-1	0.81 (0.19)	4	4 (0)

BAC, benzalkonium chloride; CHX chlorhexidine digluconate; MIC, minimum inhibitory concentration; MBC, minimum bactericidal concentration.

**Detection of*norA*and*qac*genes**.

The *norA* gene was detected by PCR in all *S. lugdunensis* strains (Table 2). Two strains were positive for the *qacA* gene. Interestingly, these two strains had decreased susceptibility to both antiseptics (CHX and BAC) (Table 2) and belonged to CC3. The *qacC, qacG, qacH* and *qacJ* genes were not detected. To rule out false negative PCR in the highly variable *qac* genes, their absence was confirmed by blastn in the 11 strains whose genome sequences were available (SL_13, SL_29, SL_55, SL_117, SL_118, SL_122, 22FJ, 25AC, 27HJ, 33RM, 37BH).

## Discussion

*S. lugdunensis* is recognized as a virulent species of CoNS responsible for severe infections. In hospital settings, predominant clones (CC1 and CC3) have been described [5, 6, 8], suggesting a potential adaptation to this environment. With the widespread use of antiseptics in healthcare facilities, there are concerns about antiseptic tolerance and resistance [17]. However, little is known about *S. lugdunensis* susceptibility to antiseptics.

Only two previous studies on antiseptic susceptibility have included a small number of *S. lugdunensis* strains: one in Sommer et al.’s work [22] and eight in the study of Addetia et al. [20]. Our study is the first to investigate the antiseptic susceptibility of such a large collection of *S. lugdunensis* strains from different clinical settings and geographical origins and representing the seven CCs described to date. Resistance was assessed by determining MIC and MBC by broth microdilution for the two commonly used antiseptics CHX and BAC. Standardized methods to determine MIC/MBC and consensus to define antiseptic resistance are missing [23]. Therefore, we applied protocols and MIC breakpoints widely used in literature for *Staphylococcus spp*. and defined reduced susceptibility to CHX and BAC when MIC > 1.5 mg/L and > 3 mg/L, respectively [18].

In our study, lower BAC MICs were observed (0.25 mg/L to 8 mg/L) compared to previous studies using broth microdilution. Indeed, they have shown that BAC MICs for clinical CoNS strains and *S. aureus* varied similarly between 0.25 mg/L and 64 mg/L [24–28]. The only two *S. lugdunensis* strains with reduced susceptibility to BAC in our study belonged to the CC3. In contrast, the CC5 and singleton ST strains tended to have lower MICs. For *S. aureus*, Kernberger-Fisher et al. have similarly showed that strains belonging to the major human lineages CC22 and CC5 had significantly higher CHX MIC values than the main animal lineage ST398 [29].

*S. lugdunensis* strains exhibited here low CHX MICs (MIC_90_ = 2 mg/L) with a narrow value distribution, similar to that obtained by Addetia et al. for eight *S. lugdunensis* strains (CHX MICs determined by broth microdilution ranging from 0.5 to 1 mg/L) [20]. In contrast, in literature, CHX MICs vary from 0.125 mg/L to 32 mg/L for other CoNS species [20, 24, 25, 28, 30], and from 0.25 mg/L to 8 mg/L for *S. aureus* [24, 25].

The CHX and BAC MBC determined here were close to MIC values, and remained much below the antiseptic concentration used in practice. Considering the epidemiological cut-off values (ECOFFs) defined for *S. aureus* by Morissey et al. (i.e. MIC = 8 mg/L and MBC > 64 mg/L for CHX, and MIC = 16 mg/L and MBC = 32 mg/L for BAC), all the 49 *S. lugdunensis* strains tested would be considered as wild-type [31].

In addition, resistance to CHX and BAC was assessed by a genotypic method. The *norA* gene was detected in all strains of our collection, suggesting that it belongs to the core genome of *S. lugdunensis*. Similarly, Costa et al. showed that *norA* is part of the core genome of *S. aureus*, but exists as multiple alleles [19]. Increased antiseptic resistance of *S. aureus* strains has been associated with NorA-mediated efflux via the overexpression of the *norA* gene [32, 33]. Thus, it would be interesting to compare *norA* expression levels of the strains in our collection.

On the contrary, the prevalence of the *qacA* gene was low (4.1%) in this study. This result contrasts with the variations of the *qacA* prevalence previously described for CoNS (42.4–62.4%) [24, 25, 34] and for *S. aureus* (from 10.5 to 21.8% for methicillin-susceptible strains and from 8,3 to 83.3% for methicillin-resistant strains) [18, 24, 25, 34–36]. Here, the *qacC*, *qacG, qacH* and *qacJ* genes were not detected by PCR. Due to lack of DNA conservation, some of the PCR primers described in the literature may fail to detect *qac* genes [14]. However, blast analysis confirmed the absence of *qac* gene sequences in the 11 whole genome sequences available. The prevalence of these genes in other CoNS species and in *S. aureus* varies greatly between studies (0–44.2% of isolates) [18, 24, 34–36]. The low prevalence of these plasmid-encoded *qac* genes observed for *S. lugdunensis* could be explained by the multiple barriers of this species genome that prevent horizontal gene transfer by mobile genetic elements [11, 37].

Interestingly, the two *S. lugdunensis* strains positive for the *qacA* resistance gene had reduced susceptibility for both CHX and BAC. This could suggest a relationship between *qac* genes and reduced susceptibility to antiseptics, as demonstrated in several studies for *S. aureus* and CoNs [20, 22–24] but to be confirmed on a larger number of strains. Whole genome studies of *S. lugdunensis* strains with reduced susceptibility to antiseptics could lead to identify mechanisms contributing to a reduced susceptibility to antiseptics in this species.

Cross-resistance to antibiotics and antiseptics remains controversial. Some studies have reported cross-resistance between CHX and antibiotics (e.g. cefoxitin, penicillin, ciprofloxacin, trimethoprim-sulfamethoxazole, clindamycin, tetracyclin), especially for *S. epidermidis*, *S. warneri* and *S. aureus* [25, 38, 39]. The only MRSL strain in our study showed reduced susceptibility to both CHX and BAC. MRSL, more prevalent in Asia [9, 10, 40], could represent a major health issue worldwide. Analysis of a larger number of MRSL strains would therefore be necessary to track such a putative link between methicillin resistance and reduced susceptibility to antiseptics.

## Conclusions

This study conducted on a large collection of strains shows that, unlike other CoNS, *S. lugdunensis* is highly susceptible to CHX and BAC. However, the first description of a reduced susceptibility to these antiseptics in two CC3 strains, highlights a potential risk for infection control in healthcare settings.

## Materials and methods

### Bacterial strains

Forty-nine clinical strains of *S. lugdunensis* isolated from 49 patients were included in this study [see Additional file 1]. They were recovered from carriage (*n* = 12) or infections (*n* = 37) and collected in seven French regions (Strasbourg, Rouen, Tours, Nancy, Montpellier, Nantes, Bordeaux) and Sweden (one strain from Kronoberg) between 2013 and 2016. All 49 strains were identified by matrix-assisted laser desorption/ionization time-of-flight (MALDI-TOF) mass spectrometry (Bruker Daltonik GmbH, Bremen, Germany). Strains were previously characterized by MLST and/or *fbl*-typing [5]. Forty-five strains belonged to the seven CCs described [(CC1, *n* = 11), (CC2, *n* = 5), (CC3, *n* = 11), (CC4, *n* = 4), (CC5, *n* = 5), (CC6, *n* = 5) and (CC7, *n* = 4)], three were singleton STs [(ST13, *n* = 1) and (ST28, *n* = 2)] and for one strain data were not obtained. *S. aureus* ATCC 25923 was used as a quality control strain for all the susceptibility tests [20, 41].

### Antibiotic susceptibility testing

Antimicrobial susceptibility was assessed by the disk diffusion method on Mueller-Hinton agar (Bio-Rad, Marnes-la-Coquette, France) according to the European Committee on Antimicrobial Susceptibility Testing recommendations (https://www.eucast.org/) [41]. The following antibiotic disks were tested: penicillin G (1 UI), kanamycin (30 µg), tobramycin (10 µg), gentamicin (10 µg), erythromycin (15 µg), clindamycin (2 µg), quinupristin/dalfopristin (15 µg), cefoxitin (30 µg), norfloxacin (10 µg), trimethoprim/sulphamethoxazole (25 µg), fusidic acid (10 µg), rifampicin (5 µg), levofloxacin (5 µg), tetracyclin (30 µg), minocyclin (30 µg), and linezolid (10 µg) (I2A, Montpellier, France).

### Minimum inhibitory concentration and minimal bactericidal concentration determination

The MIC of CHX (Sigma-Aldrich, Saint Louis, USA) and BAC (Sigma, Saint Louis, USA) was determined by broth microdilution method according to the Clinical and Laboratory Standards Institute guidelines [42]. The concentration range tested for CHX and BAC varied from 0.25 mg/L to 128 mg/L. Strains with MIC below or equal to 1 mg/L were categorized susceptible to CHX ; reduced susceptibility was defined for strains with MIC between 1.5 mg/L and 3 mg/L. Strains with MIC below or equal to 3 mg/L were considered susceptible to BAC and strains with MIC values higher than 3 mg/L exhibited reduced susceptibility [18].

For MBC determination, 10 µL of the suspension in MIC wells without visible microbial growth were subcultured onto Mueller-Hinton agar and incubated at 37 °C for 24 h. The MBC was noted as the lowest antiseptic concentration for which no growth was observed [33].

All MIC and MBC experiments were carried out in triplicate. MIC and MBC obtained twice were recorded as the final values.

### Molecular detection of antiseptic resistance genes

DNA extraction was performed using the InstaGene™ Matrix kit (Bio-Rad), according to the manufacturer’s recommendations. Each strain’s DNA was screened by PCR for the presence of *norA, qacA, qacC, qacG, qacH*, and *qacJ* genes using primers presented in Table 4 [43]. PCR reaction was composed of 0.25 µL (0.50 µM) of each primer, 12.5 µL of Go Taq® G2 Green Master Mix, 5 µL of DNA and 7 µL of sterile water, for a final volume of 25 µL. PCRs were performed, using a Veriti Thermal Cycler (Applied Biosystems, Foster City, CA, USA), as follows: initial denaturation step at 94 °C for 3 min, 30 cycles of 94 °C for 1 min, 50 °C for 1 min, 72 °C for 1 min, and final extension step at 72 °C for 5 min. PCR products were visualized under UV after migration for 45 min at 110 V on 1.5% gel containing 0.005 mg/L ethidium bromide.

**Table 4:** List of PCR primers

Primer	Target gene	Primer Sequence (5’-3’)	Product size (pb)	Reference
norA1_F norA1_R	*norA*	TTTGTCACGCTTATCATTTCA CCCGTCTGTTGTTTGTTG	291	This study
norA2_F norA2_R	*norA*	TGCGATTGTGGGTGGAGG TCCAATAACCGTTTGCCAAGA	155	This study
qacA_F qacA_R	*qacA*	GCAGAAAGTGCAGAGTTCG CCAGTCCAATCATGCCTG	361	Noguchi et al.1999
qacC_F qacC_R	*qacC*	GCCATAAGTACTGAAGTTATTGGA GACTACGGTTGTTAAGACTAAACCT	195	Noguchi et al.1999
qacG_F qacG_R	*qacG*	CAACAGAAATAATCGGAACT TACATTTAAGAGCACTACA	275	Ignak et al. 2017
qacH_F qacH_R	*qacH*	ATAGTCAGTGAAGTAATAG AGTGTGATGATCCGAATGT	295	Ignak et al. 2017
qacJ_F qacJ_R	*qacJ*	CTTATATTTAGTAATAGCG GATCCAAAAACGTTAAGA	306	Ignak et al. 2017

**Research of*qac*genes in whole genomes**.

We collected the 11 whole genomes of strains used in this study available on NCBI (accession numbers: GCA_008728755.1, GCA_008728775.1, GCA_008728795.1, GCA_008728815.1, GCA_008728715.1, GCA_008728735.1, GCA_002097035.1, GCA_002096135.1, GCA_002096155.1, GCA_002104555.1, GCA_002096075.1) and the reference sequences of the *qacA*, *qacC*, *qacG*, *qacH* and *qacJ* genes (NCBI accession numbers: NC_007931.1, GQ900464.1, NG_051904.1, NC_019081.1, NG_048046.1) cited in the publication by Worthing KA et al., 2018 [44]. Each *qac* gene reference sequence was searched for in the whole genomes of the strains using blastn.

### Electronic supplementary material

Below is the link to the electronic supplementary material.

Supplementary Material 1

## Data Availability

All data of this study are included in this article [and in its supplementary information file].

## Abbreviations

BAC: Benzalkonium chloride
CC: Clonal complex
CHX: Chlorhexidine digluconate
CoNS: Coagulase-negative staphylococci
DNA: Desoxyribonucleic acid
ECOFF: Epidemiological cut-off
MBC: Minimum bactericidal concentration
MIC: Minimum inhibitory concentration
MLST: Multilocus sequence typing
MRSL: Methicillin-resistant *S. lugdunensis*
PCR: Polymerase chain reaction
QAC: Quaternary ammonium compound
