# Supplementary material for: Impact of clonal lineages on susceptibility of Staphylococcus lugdunensis to chlorhexidine digluconate and chloride benzalkonium
Source: BMC Microbiol. 2023 Nov 13;23:337. doi: 10.1186/s12866-023-03088-1 (PMC10642039; doi:10.1186/s12866-023-03088-1)
Supplement: Supplementary file 1 — Supplementary Material 1 [file 12866_2023_3088_MOESM1_ESM.docx]

**Additional files**

**Additional file 1.** Characteristics of the 49 clinical strains of *S. lugdunensis* studied

| **Strain** | **Clonal complex CC** | **Sequence typing (ST)** | **City** | **Clinical sources / sampling** | **Community-acquired infection (C)/Nosocomial infection (N)** | **Date of collection** | **Resistance genes** | **CHX** | | | **BAC** | |
| --- | --- | --- | --- | --- | --- | --- | --- | --- | --- | --- | --- | --- |
|  |  |  |  |  |  |  |  | **MIC** | | **MBC** | **MIC** | **MBC** |
| 57FJM | 1 | 1 | Strasbourg | Infection | N | 2015.06.03 | *norA* | 1 | 4 | | 2 | 4 |
| **SL_29** | **1** | **1** | **Rouen** | **Infection** | **ND** | **ND** | ***norA*** | **2** | 4 | | 2 | 4 |
| SL_122 | 1 | 1 | Tours | Carriage | ND | ND | *norA* | 1 | 2 | | 2 | 4 |
| 37BH | 1 | 1 | Strasbourg | Infection | C | 2014.12.04 | *norA* | 1 | 2 | | 1 | 4 |
| SL_117 | 1 | 1 | Kronoberg | Carriage | ND | ND | *norA* | 1 | 2 | | 2 | 4 |
| **07SM** | **1** | **6** | **Strasbourg** | **Infection** | **N** | **2014.02.24** | ***norA*** | **2** | 4 | | 1 | 4 |
| 33RM | 1 | 6 | Strasbourg | Infection | N | 2014.11.03 | *norA* | 1 | 2 | | 1 | 4 |
| SL_13 | 1 | 6 | Rouen | Infection | ND | ND | *norA* | 1 | 4 | | 2 | 4 |
| **SL_74** | **1** | **12** | **Nancy** | **Infection** | **ND** | **ND** | ***norA*** | **2** | 2 | | 1 | 4 |
| C60 | 1 | 12 | Strasbourg | Carriage | N | 2014.11.27 | *norA* | 1 | 2 | | 1 | 4 |
| 27BC | 1 | 12 | Strasbourg | Infection | C | 2014.10.15 | *norA* | 1 | 2 | | 1 | 2 |
| 74KR | 2 | 2 | Strasbourg | Infection | C | 2015.10.23 | *norA* | 1 | 4 | | 1 | 4 |
| SL_85 | 2 | 2 | Montpellier | Infection | ND | ND | *norA* | 1 | 4 | | 2 | 4 |
| SL_56 | 2 | 14 | Nantes | Infection | ND | ND | *norA* | 1 | 4 | | 2 | 4 |
| SL_73 | 2 | 19 | Nancy | Infection | ND | ND | *norA* | 1 | 2 | | 2 | 2 |
| 64865028 | 2 | ND | Rouen | Carriage | ND | ND | *norA* | 1 | 4 | | 1 | 4 |
| **C47** | **3** | **3** | **Strasbourg** | **Carriage** | **N** | **2014.10.07** | ***norA/qacA*** | **2** | 4 | | **4** | 8 |
| C85 | 3 | 3 | Strasbourg | Carriage | C | 2015.06.15 | *norA* | 1 | 4 | | 1 | 4 |
| 50SD | 3 | 3 | Strasbourg | Infection | C | 2015.04.02 | *norA* | 1 | 2 | | 1 | 4 |
| 31FE | 3 | 3 | Strasbourg | Infection | C | 2014.11.15 | *norA* | 1 | 4 | | 1 | 4 |
| C08 | 3 | 3 | Strasbourg | Carriage | C | 2013.11.05 | *norA* | 1 | 4 | | 2 | 4 |
| C27 | 3 | 3 | Strasbourg | Carriage | N | 2014.01.22 | *norA* | 1 | 2 | | 2 | 8 |
| 03MC | 3 | 3 | Strasbourg | Infection | N | 2013.12.28 | *norA* | 1 | 2 | | 1 | 4 |
| 04SZ | 3 | 3 | Strasbourg | Infection | N | 2013.11.14 | *norA* | 1 | 2 | | 1 | 4 |
| 76LE | 3 | 3 | Strasbourg | Infection | N | 2015.11.09 | *norA* | 1 | 4 | | 1 | 4 |
| 16060354 | 3 | ND | Rouen | Carriage | ND | ND | *norA* | 1 | 2 | | 2 | 4 |
| **SL_RM** | **3** | **3** | **Rouen** | **Infection** | **ND** | **ND** | ***norA/qacA*** | **2** | 2 | | **8** | 8 |
| 34SM | 4 | 4 | Strasbourg | Infection | C | 2014.11.05 | *norA* | 1 | 4 | | 1 | 4 |
| 48SA | 4 | 4 | Strasbourg | Infection | N | 2015.03.25 | *norA* | 1 | 2 | | 1 | 4 |
| SL_62 | 4 | 17 | Bordeaux | Infection | ND | ND | *norA* | 1 | 4 | | 2 | 8 |
| 16084449 | 4 | ND | Rouen | Infection | N | ND | *norA* | 1 | 2 | | 2 | 4 |
| 25AC | 5 | 5 | Strasbourg | Infection | C | 2014.09.15 | *norA* | 1 | 2 | | 0.5 | 4 |
| **73KM** | **5** | **5** | **Strasbourg** | **Infection** | **C** | **2015.10.22** | ***norA*** | **2** | 2 | | 0.5 | 4 |
| SL_66 | 5 | 18 | Bordeaux | Infection | ND | ND | *norA* | 1 | 4 | | 1 | 4 |
| 16035553 | 5 | ND | Rouen | Infection | N | ND | *norA* | 1 | 2 | | 0.25 | 2 |
| 16103574 | 5 | ND | Rouen | Infection | C | ND | *norA* | 1 | 4 | | 0.5 | 4 |
| SL_118 | 6 | 10 | Kronoberg | Carriage | ND | ND | *norA* | 1 | 4 | | 1 | 4 |
| SL_55 | 6 | 10 | Nantes | Infection | ND | ND | *norA* | 1 | 4 | | 2 | 4 |
| SL_DSM 4804 | 6 | 24 | Lyon | Infection | ND | ND | *norA* | 1 | 2 | | 1 | 4 |
| 22FJ | 6 | 24 | Strasbourg | Infection | C | 2014.08.09 | *norA* | 1 | 2 | | 2 | 8 |
| 16084028 | 6 | ND | Rouen | Infection | N | ND | *norA* | 1 | 4 | | 2 | 8 |
| 77HJ | 7 | 26 | Strasbourg | Infection | N | 2015.11.11 | *norA* | 1 | 4 | | 2 | 4 |
| C42 | 7 | 26 | Strasbourg | Carriage | N | 2014.07.27 | *norA* | 1 | 4 | | 2 | 8 |
| 70GR | 7 | 27 | Strasbourg | Infection | N | 30.09.2015 | *norA* | 1 | 2 | | 1 | 4 |
| 16043959 | 7 | ND | Rouen | Infection | C | ND | *norA* | 1 | 4 | | 1 | 4 |
| SL_10 | Singleton | 13 | Rouen | Infection | ND | ND | *norA* | 1 | 4 | | 1 | 4 |
| 65KN | Singleton | 28 | Strasbourg | Infection | N | 2015.08.18 | *norA* | 1 | 2 | | 1 | 4 |
| 69MP | Singleton | 28 | Strasbourg | Infection | C | 2015.09.23 | *norA* | 1 | 4 | | 1 | 4 |
| 16116446 | ND | ND | Rouen | Carriage | ND | ND | *norA* | 0.5 | 2 | | 0.25 | 4 |

ND: No Data

Strains with a reduced susceptibility are in bold text
